# Dynein-Dynactin Complex Is Essential for Dendritic Restriction of TM1-Containing *Drosophila* Dscam

**DOI:** 10.1371/journal.pone.0003504

**Published:** 2008-10-23

**Authors:** Jacob Shun-Jen Yang, Jia-Min Bai, Tzumin Lee

**Affiliations:** 1 Department of Neurobiology, University of Massachusetts Medical School, Worcester, Massachusetts, United States of America; 2 Neuroscience Program, University of Illinois at Urbana-Champaign, Urbana, Illinois, United States of America; The University of Queensland, Australia

## Abstract

**Background:**

Many membrane proteins, including *Drosophila* Dscam, are enriched in dendrites or axons within neurons. However, little is known about how the differential distribution is established and maintained.

**Methodology/Principal Findings:**

Here we investigated the mechanisms underlying the dendritic targeting of Dscam[TM1]. Through forward genetic mosaic screens and by silencing specific genes via targeted RNAi, we found that several genes, encoding various components of the dynein-dynactin complex, are required for restricting Dscam[TM1] to the mushroom body dendrites. In contrast, compromising dynein/dynactin function did not affect dendritic targeting of two other dendritic markers, Nod and Rdl. Tracing newly synthesized Dscam[TM1] further revealed that compromising dynein/dynactin function did not affect the initial dendritic targeting of Dscam[TM1], but disrupted the maintenance of its restriction to dendrites.

**Conclusions/Significance:**

The results of this study suggest multiple mechanisms of dendritic protein targeting. Notably, dynein-dynactin plays a role in excluding dendritic Dscam, but not Rdl, from axons by retrograde transport.

## Introduction

Neurons exhibit highly polarized structures, including two morphologically and functionally distinct domains, axons and dendrites. Dendrites and axons respectively receive or send information, proper execution of which requires different sets of molecules. For example, in the mammalian brain and in cultured neurons, voltage-gated potassium channels of the Kv1 (*Shaker*) family reside in the axons. In contrast, voltage-gated potassium channel Kv2.1 and Kv2.2 are selectively enriched in the somatodendritic region [1]–[3]. The dendritic potassium channels undergo slower inactivation to prevent back-propagation of action potentials into the dendrites [4], [5]. Certain metabotropic glutamate receptors, including mGluR1a and mGluR2, also show polarized distribution [6], and potentially underlie differential glutamate effects in different compartments of neurons [7], [8].

One dominant model to explain the differential distribution of neuronal membrane proteins involves directed transport of vesicular cargos along the microtubules that extend into the dendrites and axons [9]. Microtubules have polarity; directed transport requires motors to move cargos toward the plus- or minus-end of the microtubules. In axons microtubules are uniformly oriented with minus-ends pointing to the cell body, while microtubules exist with mixed polarity within the somatodendritic region [10]. This difference in microtubule organization supports the hypothesis that minus-end-directed motors are constantly moving molecules out of axons and may selectively transport their cargos into the dendrites [11]. Identified minus-end-directed motors include dynein and C-terminal kinesins. Cytoplasmic dynein, which forms a large complex with its activator dynactin, is responsible for the retrograde transport in axons [12], [13]. Dynein/dynactin complex contains more than twenty subunits. Although the functions of each subunit remain to be determined, it is believed that all the subunits act together to regulate the processivity and cargo-binding selectivity of dynein [13], [14]. Various C-terminal kinesins (i.e. *Ncd* in *Drosophila* and *KIFC2* in *mouse*), which carry their motor domain at the C-terminus, also move specifically toward the minus end. But their real function in vesicular transport is unclear [15], [16]. The role of minus-end-directed motors in dendritic protein targeting remains undocumented.

Besides selective transport, additional mechanisms may contribute to the polarized distribution by differential depletion or stabilization. For example, the steady-state axonal distribution of Nav1.2 and VAMP2 is primarily achieved through their selective removal by endocytosis from the dendritic plasma membrane [17], [18]. Preferential fusion of vesicular cargos with different plasma membrane domains may mediate some polarized distribution as well. One precedent for fusion selectivity involves targeting of distinct SNAREs to the apical or basolateral domains of epithelial MDCK cells [19]. Other possible mechanisms include existence of diffusion barriers and/or protein stabilization by scaffold proteins. However, most of these studies shed light on the polarized distribution of axonal proteins; and little is known about dendritic protein targeting [6], [20], [21].

*Drosophila* Down Syndrome cell adhesion molecule (Dscam) is a transmembrane protein, which belongs to the immunoglobulin (Ig) superfamily. Dscam is essential for diverse neuronal morphogenetic processes, including axon guidance, branch segregation, and dendritogenesis [22]–[25]. Notably, *Drosophila Dscam* can encode thousands of isoforms through alternative splicing involving many choices of exon 4, 6, 9 and 17. Distinct Dscam isoforms may be targeted to dendrites or axons, depending on which of the two transmembrane-domain-encoding exon 17 alternatives, 17.1 or 17.2, is utilized [26]. Dscam isoforms carrying exon 17.1 (Dscam[TM1]) are largely restricted to dendrites, while Dscam isoforms with exon 17.2 (Dscam[TM2]) are enriched in axons. Further, depleting Dscam[TM1] or Dscam[TM2] blocks morphogenesis of dendrites versus axons [27]. Understanding how isoforms of Dscam are differentially distributed in neurons promises to shed new light on neuron polarity and its underlying mechanisms.

Here we performed genetic mosaic screens to identify genes required cell-autonomously for the dendritic targeting of Dscam[TM1]. We obtained mutants that exhibit different mislocalization phenotypes. We identified three mutations in the known components of dynein-dynactin complex (*Lis1*, *p24* and *Dynamitin*) that all affect Dscam dendritic targeting. Misdistribution of dendritic Dscam to axons was also observed when we suppressed the expression of other dynein/dynactin components with RNA interference. However, microtubule polarity in the mutant axons was maintained. Transient induction of Dscam[TM1] further revealed that disrupting dynein/dynactin function did not affect the targeting of newly synthesized Dscam[TM1] to the dendrites. Instead, dendritic Dscam later diffused into the axons. These observations indicate that dynein/dynactin plays a role in maintaining dendritic restriction of Dscam[TM1], and further suggest a dynein/dynactin-independent mechanism for the initial targeting of Dscam[TM1] to dendrites. Notably, dynein/dynactin dysfunction did not alter distribution of another dendritic transmembrane protein Rdl (*Resistant to Dieldrin*), supporting involvement of diverse mechanisms in locating distinct molecules to the dendritic membrane.

## Results

### *Drosophila* Dscam[TM1] as a dendritic marker for genetic mosaic analysis of dendritic protein targeting

**Figure 1:**
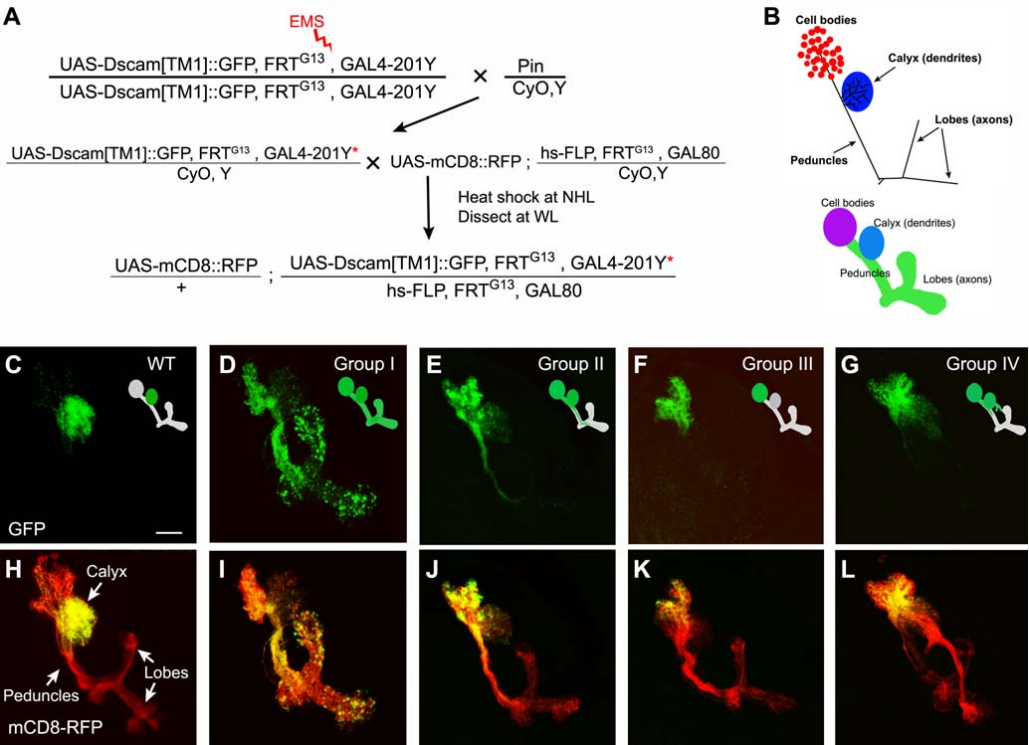
Genetic mosaic screen for mutants with abnormal Dscam[TM1] distribution. (A) Schemes of the genetic crosses of the screen. The star represents a mutagenized chromosome. (B) Schematic diagram of MB subcompartments. (C–L) Composite confocal images of MB neuroblast clones co-labeled with mCD8::RFP (red) and Dscam[TM1]::GFP (green). As compared to the wild-type control (C) where transgenic Dscam was absent from axons, various mutant clones (D, E, F, and G) exhibited different Dscam mislocalization phenotypes. Note that mutations of group IV disrupted MB gross morphologies (G and L) and were all mapped to the gene *short-stop*. Scale bar (here and in all figures) represents 20 µm.

We have previously shown that transgenic Dscam carrying the exon 17.1-encoding transmembrane domain (referred to as Dscam[TM1] as opposed to Dscam[TM2] that carries exon 17.2) is selectively targeted to dendrites. When ectopically expressed in the neurons of the *Drosophila* olfactory learning and memory center, the mushroom bodies (MBs), Dscam[TM1]::GFP exists abundantly in the calyx where MB dendrites are located, but could not be detected in the axons which extend through the peduncle before entering the MB lobes (Figures 1B, 1C and 1H). MARCM, a positive-labeling genetic mosaic technique, has allowed us to effectively generate clones of MB neurons that are homozygous for a specific chromosome arm in an otherwise heterozygous organism and simultaneously express a reporter gene in an unlabeled background [28], [29]. Using mCD8::GFP as a reporter to visualize the morphology of the MBs, we have been screening for genes required for various aspects of MB development through loss-of-function genetic mosaic analysis [25], [30]–[34]. We reasoned that incorporating Dscam[TM1]::GFP into our MARCM screens should allow us to uncover genes, regardless of their possible involvement in other essential cellular events, that are essential for proper dendritic targeting of Dscam[TM1]::GFP. Our goal was to fully elucidate the cellular/molecular mechanisms of dendritic protein targeting.

To adapt the system for genetic mosaic screens on dendritic protein targeting, we incorporated *UAS-mCD8::RFP* and *UAS-Dscam[TM1]::GFP* into MARCM (Figure 1A). In combination with *GAL4-201Y*, a MB GAL4 enhancer trap line, we simultaneously expressed Dscam[TM1]::GFP and mCD8::RFP in the MB clones and directly examined Dscam[TM1]::GFP distribution inside the MBs of live mosaic larval brains (Figure 1C). While mCD8::RFP outlined the entire clone (Figure 1H, red), Dscam[TM1]::GFP was well restricted to the MB calyx in wild-type clones (Figure 1C). Using this as readout, we screened 1,850 chemically mutagenized 2R chromosome arms for mutations that affect the dendritic restriction of Dscam[TM1]::GFP. We recovered 35 mutant lines that exhibited abnormal Dscam[TM1]::GFP protein distribution patterns. We clustered them into four groups according to their phenotypes. Group I consisted of 9 independent lines that showed significant Dscam[TM1]::GFP accumulation in both MB peduncles and lobes (e.g. Figures 1D and 1I). Group II carried mutations that have mistargeted Dscam[TM1]::GFP gradually disappeared along the MB axon bundles (e.g. Figures 1E and 1J). In Group III, Dscam[TM1]::GFP becomes restricted to MB cell bodies (e.g. Figures 1F and 1K), while mutations in group IV disrupted gross MB morphology (e.g. Figures 1G and 1L). All the recovered lines were lethal as homozygotes, thus it would be impossible to systematically uncover the genes required for dendritic protein targeting as well as organism viability without genetic mosaics. In the following work, we selectively focused on group I mutants that displayed mistargeting of dendritic Dscam more uniformly throughout the MBs.

### Analysis of mutants that exhibited aberrant accumulation of Dscam[TM1]::GFP in axons

**Figure 2:**
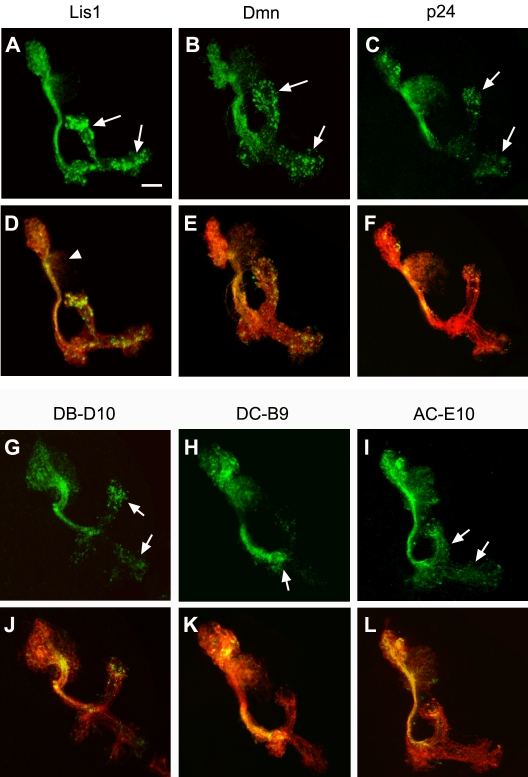
Mistargeting of dendritic Dscam in Group I mutant clones. MB clones of different complementation groups of group I. Granular accumulation of Dscam[TM1]::GFP (green) in the MB lobes was observed in four of the six complementation groups, including *Lis1*, *Dmn*, *p24* and DB-D10 (A–C and G, arrows). In contrast, mistargeted Dscam preferentially accumulated in the peduncles of DC-B9 mutant clones (H, arrow), while Dscam[TM1]::GFP was rather uniformly distributed in AC-E10 clones (I, arrows). MB clones were co-labeled by mCD8::RFP (red). Note the reduced dendritic region in *Lis1* mutant clone (arrowhead).

Detailed analysis of group 1 mutants further revealed subclasses of misdistribution phenotypes. Five of the nine mutants exhibited granular accumulation of Dscam[TM1]::GFP in the MB lobes (e.g. Figures 2A–2C and 2G), three had Dscam[TM1]::GFP selectively accumulated in the peduncle (e.g. Figure 2H), and the last one showed broad non-granular distribution of Dscam[TM1]::GFP (e.g. Figure 2I). In addition, many of the mutant clones were smaller than controls (e.g. Figure 1H). Two of the lines with granular accumulation had reduced calycal volume, suggesting possible defects in dendritic morphogenesis. These phenomena indicate that genes involved in dendritic protein targeting potentially underlie multiple fundamental cellular functions. Further, the identification of several clusters of misdistribution phenotypes suggests the involvement of multiple mechanisms in restricting Dscam[TM1] to dendrites. However, distinct phenotypes might simply result from allele variations in genes of similar function.

Complementation among the mutations yielded six complementation groups. Mapping against deficiency lines and other known mutations further revealed that mutations in *Lis1*, *p24*, and *Dynamitin (Dmn)* constituted three of the four complementation groups which showed mistargeted Dscam[TM1]::GFP in granules (Figures 2A–2C, arrows). Both lines that exhibited defective dendritic morphogenesis carried mutations in *Lis1* (Figure 2D, arrowhead). *Lis1*, a mutation of which underlies human *lissencephaly*, is a regulatory protein of the microtubule motor dynein, and is highly conserved from human to *Drosophila*. *Drosophila Lis1* has been shown to play an essential role in MB neurogenesis and dendritic elaboration [35]–[37]. However, it has never been shown to be involved in differential distribution of cell surface proteins. *p24 (CG9893)* is a novel molecule that may be integral to the dynactin complex, as implicated from its sequence and structural similarity with vertebrate *DCTN3* [12]. *p50/Dmn* is also a dynactin subunit. The dynactin complex regulates the cargo selection and processivity of dynein. Mutations in the dynactin complex can affect the assembly of dynein/dynactin complex and its binding affinity for microtubules [13], [38], [39]. The recovery of multiple dynein/dynactin components and regulators indicates that proper dynein/dynactin function is essential for the restriction of Dscam[TM1]::GFP to dendrites.

### Requirement of dynein-dynactin complex for the restriction of Dscam[TM1]::GFP to dendrites

**Figure 3:**
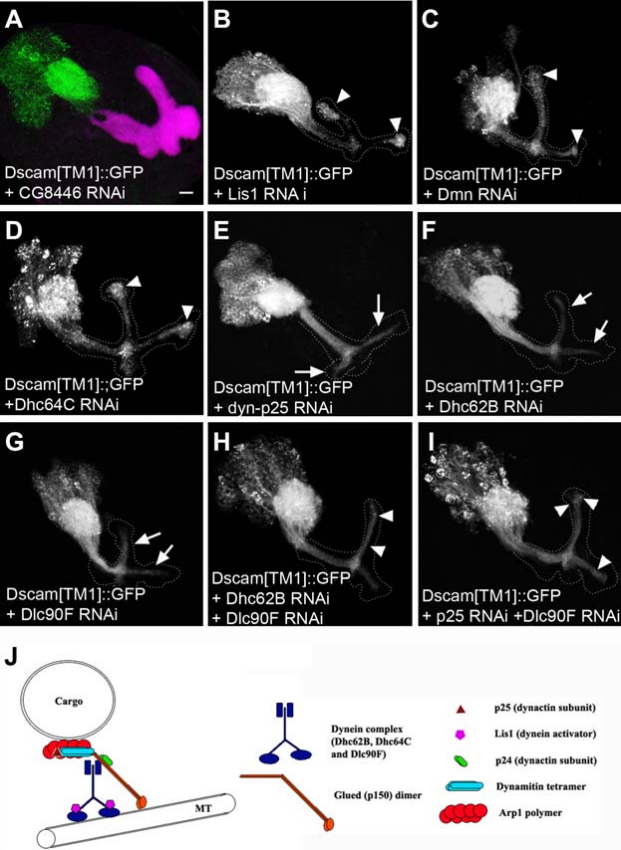
Mistargeting of dendritic Dscam following depletion of various components of dynein-dynactin complex. (A–G) Distribution of Dscam[TM1]::GFP in the larval MBs where a dynein/dynactin-unrelated gene *CG8446* (A) or various components of dynein/dynactin complex (B–G) were silenced by induction of *RNAis* with GAL4-OK107. Dscam[TM1]::GFP was no longer restricted to the cell bodies and calyx, when dynein/dynactin components were knocked down (B–G, compared to A). Note granular accumulation at the ends of axon lobes in [B] to [D] (arrowheads) versus uniform distribution in [E] to [G] (arrows). Double knockdown (H and I) showed more granular accumulation at the ends of axons than individual knockdowns (E–G) have. (J) Schematic illustration of dynein/dynactin complex. The entire axonal lobes were outlined by dashed lines according to the 1D4 mAb staining (red in A).

In order to substantiate the involvement of dynein-dynactin complex, we first confirmed that Lis1, Dmn, and p24 are required for the dendritic restriction of Dscam[TM1]::GFP using reagents independent of our genetic screen. Genes could be effectively silenced in the MBs by RNA interference (RNAi) [27], [40]; and transgenic flies carrying *UAS-RNAi* against various *Drosophila* genes, including *Lis1*, *Dmn*, and many other components of dynein-dynactin complex, are available in the Vienna Drosophila RNAi Center (VDRC) [41]. Encouragingly, silencing *Lis1* or *Dmn*, as opposed to various control genes (such as *CG8446* and *CG18247*), by targeted RNAi effectively mislocalized transgenic Dscam[TM1]::GFP to MB axon lobes (Figures 3A–3C). These results not only confirmed the roles of *Lis1* and *Dmn*, but also illustrated the utility of RNAi in quickly uncovering more genes in a common pathway. We confirmed the indispensability of *p24* in Dscam localization by examining Dscam[TM1]::GFP distribution in MB clones homozygous for a pre-existing loss-of-function allele of *p24* (data not shown). Analogous mislocalization phenotypes were obtained when Lis1, Dmn, or p24 were depleted by various means, substantiating their involvement, possibly through the dynein-dynactin complex, in excluding dendritic Dscam from axons.

Further, we knocked down additional components of the dynein/dynactin complex (Figure 3J), including dynein heavy chains (Dhc64C and Dhc62B), dynein light chain (Dlc90F), and another dynactin subunit (p25), by targeted RNAi. Aberrant accumulation of dendritic Dscam in the MB axons was detected in all the cases (Figures 3D–3G), though the detailed mislocalization patterns varied depending on which gene was silenced. For example, targeting RNAi against *Lis1*, *Dmn* or *Dhc64C* caused excessive accumulation of dendritic Dscam near the ends of the axonal lobes (Figures 3B–3D, arrowheads), while Dscam[TM1] uniformly distributed throughout the axonal lobes following depletion of *Dhc62B*, *Dlc90F* or *p25* (Figures 3E–3G, arrows). These different phenotypes could be derived from different residual dynein/dynactin function due to partial knockdown or differential redundancy. Alternatively, they might result from crippling distinct aspects of Dscam protein targeting, since it remains unclear as to the individual proteins' full spectra of function (see Discussion). Notably, simultaneously depleting either two of *Dhc62B*, *Dlc90F* or *p25* shifted the misdistribution from the peduncle to the lobes (e.g. Figures 3H and 3I), better recapitulating the terminal accumulation phenotype in other dynein/dynactin mutants. These results indicate that all these molecules act through dynein/dynactin complexes to restrict Dscam[TM1] to dendrites.

**Figure 4:**
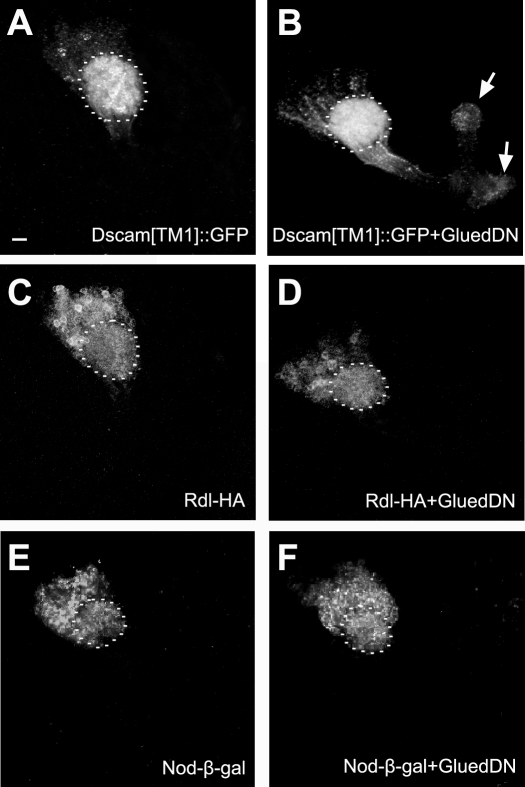
Effects of dominant-negative Glued on dendritic protein targeting. Larval MBs expressing various dendritic markers, including Dscam[TM1] (A and B), Rdl-HA (C and D) and Nod-β-gal (E and F), in the absence or presence of dominant-negative Glued. Note that dominant-negative Glued selectively affected the somatodendritic distribution of Dscam[TM1]::GFP (compare B to A), and that mislocalized Dscam[TM1]::GFP preferentially accumulated at the ends of lobes (arrows). The calyx regions were outlined by dashed lines.

In addition, the role of *Glued* was determined through inhibition of its function by a dominant-negative Glued (Gl^Δ^) [42]. Glued is the largest subunit of dynactin complex and plays a particular important role in dynein binding and enhancement of dynein processivity. Overexpression of C-terminal-truncated Glued (Gl^Δ^), known to dominantly block dynein/dynactin function, also resulted in axonal accumulation of dendritic Dscam, especially near the ends of axonal lobes (Figures 4A and 4B, arrows). These results indicate that normal dynein/dynactin function is essential for dendritic restriction of Dscam[TM1]::GFP. Suppressing any component of dynein/dynactin complex may impede dynein/dynactin function and lead to the accumulation of Dscam[TM1]::GFP in axons.

### Blocking dynein/dynactin function does not affect dendritic targeting of two other dendritic markers

To determine how broadly dynein/dynactin is involved in dendritic protein targeting, we examined whether dynein/dynactin is required for proper localization of other dendritic proteins. Several documented dendritic markers, including homer-GFP, Apc2-GFP, Act5C-GFP, Nod- β -gal and Rdl-HA [43], [44], were ectopically expressed in the larval MBs using *GAL4-201Y* as the driver. In this condition, only Nod-β-gal and Rdl-HA showed predominant somatodendritic distribution and were largely excluded from MB axon lobes (Figures 4E and 4C; data not shown). Axonal exclusion of homer-GFP and Apc2-GFP, as reported previously [43], may require a very low level of induction.

**Figure 5:**
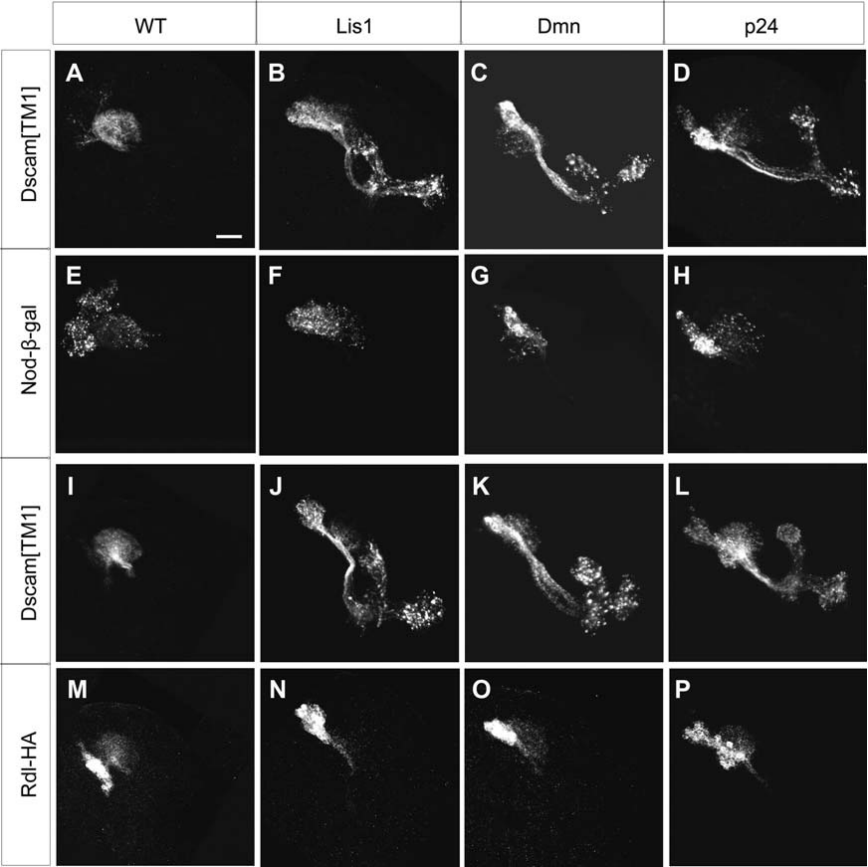
Axonal exclusion of Dscam[TM1], but not Nod or Rdl, requires dynein/dynactin. Larval MB clones co-expressing Dscam[TM1]::GFP (A–D and I–L) with Nod-β-gal (E–H) or Rdl-HA (M–P). As compared to wild-type controls, *Lis1*, *Dmn* and *p24* mutant clones had Dscam[TM1]::GFP, but not Nod-β-gal or Rdl-HA, mislocalized to the MB axons.

Nod-β-gal is a fusion protein comprised of the motor domain of Nod and β-galactosidase, and has been shown to be a reliable minus-end reporter for microtubules in *Drosophila*, including MB neurons [45]–[47]. Consistent with the notion that microtubules are uniformly oriented with plus-end pointing distally in axons, Nod-β-gal was highly enriched in dendrites and cell bodies but largely absent from peduncles and axonal lobes in wild-type MB neurons (Figure 4E and 5E) [30], [43]. Co-expression with dominant-negative Glued or ectopic induction in dynein/dynactin mutant clones (*Lis1*, *Dmn* and *p24*) did not alter its somatodendritic distribution (Figures 4F, 5F, 5G and 5H). These results suggest that dynein/dynactin dysfunction did not perturb microtubule organization in axons, and that mistargeting of Dscam[TM1]::GFP did not occur as a consequence of abnormal microtubule polarity.

Rdl-HA (*Resistant to Dieldrin*) is a GABA receptor tagged with the HA epitope, and has been shown to be well restricted to dendrites in *Drosophila* embryonic motor neurons [44]. In wild-type MB neurons, Rdl-HA was also localized in dendrites and cell bodies, and proximal region of peduncles only (Figure 4C and 5M). Again, perturbation of dynein/dynactin function using dominant-negative Glued or by MARCM with *Lis1*, *Dmn* and *p24* mutations did not alter the somatodendritic distribution of Rdl in the larval MBs (Figures 4D, 5N, 5O and 5P). These results indicate that dynein/dynactin is selectively required for exclusion of dendritic Dscam from axons, implicating utilization of different mechanisms for restricting distinct membrane proteins to the dendrites.

### Retrograde transport plays a role in maintaining but not establishing Dscam[TM1] dendritic restriction

We wondered how dynein/dynactin complexes act to ensure restriction of Dscam[TM1] to the dendrites. As a minus-end-directed microtubule motor, dynein/dynactin may actively move Dscam[TM1] from cell bodies to dendrites by selective transport. Alternatively, it may play a scavenging role and constantly remove mistargeted Dscam[TM1] out of axons via retrograde axonal transport [11], [48], [49]. To distinguish between these two possibilities, we sought to visualize newly synthesized Dscam[TM1]::GFP and examine how dynein/dynactin dysfunction might affect the initial sorting of Dscam[TM1] and/or the maintenance of its dendritic distribution.

**Figure 6:**
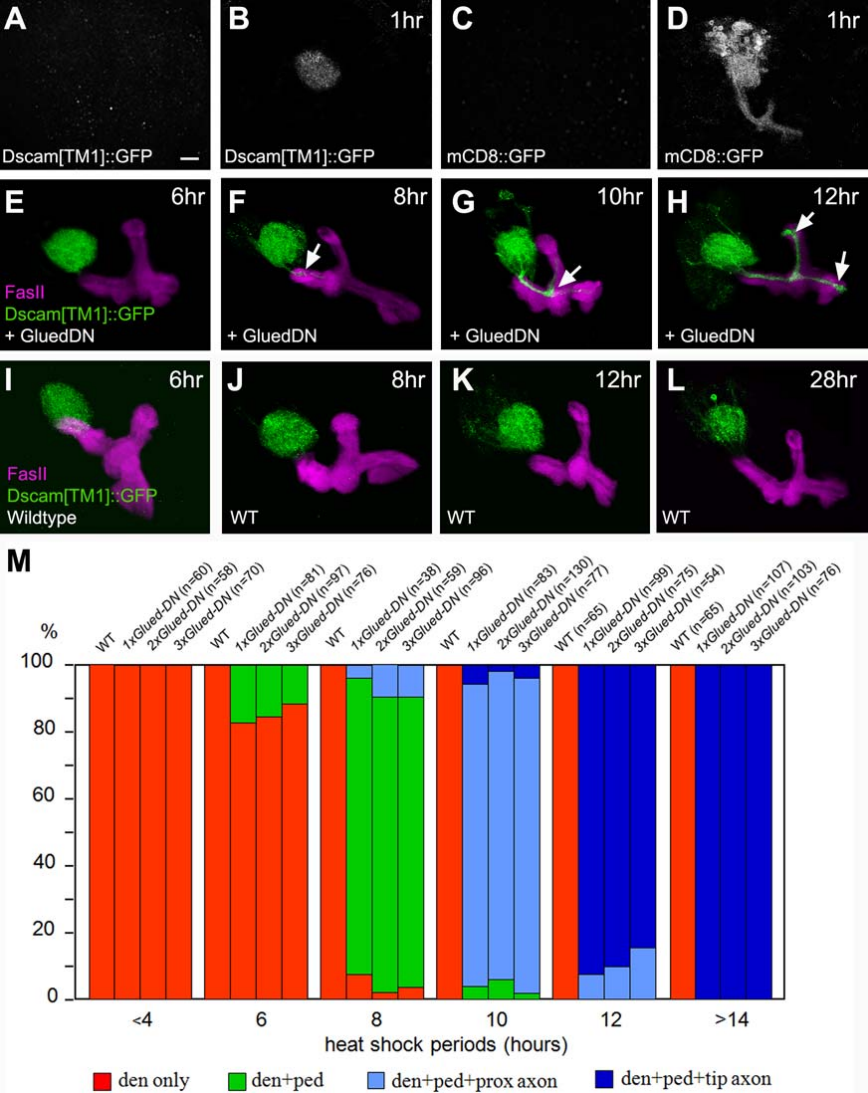
Retrograde transport plays a role in restricting Dscam[TM1]::GFP to the somatodendritic domain. (A–D) Transient induction of *UAS-Dscam[TM1]::GFP* or *UAS-mCD8::GFP* by TARGET. Prior to induction, GAL80ts fully suppressed the expression at a permissive temperature (A, C). Notably, one hour after heat-shock inactivation of GAL80ts, Dscam[TM1]::GFP was detected only in MB calyx (B) while mCD8::GFP distributed throughout the entire neurons (D). (E–L) Induction of Dscam[TM1]::GFP with or without GluedDN. Following co-induction with dominant-negative Glued, Dscam[TM1]::GFP gradually spread into MB peduncles and axonal lobes (F–H, arrows). In contrast, Dscam[TM1]::GFP was well restricted to the MB calyx in the absence of dominant-negative Glued (I–L). (M) Effects of GluedDN dosage on the misdistribution of Dscam[TM1]::GFP. Dscam[TM1]::GFP could localize in dendrites only (e.g. [E]), dendrites plus peduncles (e.g. [F]), dendrites, peduncles plus proximal portions of axon lobes (e.g. [G]), or from calyx to the tips of axon lobes (e.g. [H]). Note that increasing GlueDN dosage did not accelerate the mislocalization process. Three insertion lines of *UAS-Gl^Δ^* were examined: *UAS-Gl^Δ84^*, *UAS-Gl^Δ008m^*, and *UAS-Gl^Δ020m^*. All of them were examined individually in 1× Glued-DN. All possible combinations of them were checked in 2× Glued-DN. No statistically significant differences were detected among conditions with distinct insertions or different numbers of insertions.

Transient induction of Dscam[TM1]::GFP in the larval MBs was achieved using the TARGET system, in which GAL4-dependent expression of UAS-transgene is acutely controlled by a temperature-sensitive GAL4 repressor, GAL80ts [50]. At 18°C, GAL4-OK107 was fully suppressed by GAL80ts (Figure 6A and 6C). Following inactivation of GAL80ts by shifting the organisms to higher temperatures (see Experimental Procedures), we could start to detect mCD8::GFP or Dscam[TM1]::GFP in young MB neurons (whose axons occupy core regions of axonal bundles and are weakly labeled by 1D4 mAb [51]) approximately one hour after induction. Since the enrichment of newly synthesized protein in young MB neurons were seen for both mCD8::GFP and Dscam[TM1]::GFP, this phenomenon could possibly result from the expression profile of GAL4-OK107 at the wandering larval stage or the difference in the intrinsic properties of newly derived MB neurons versus mature ones. Notably, while mCD8::GFP was uniformly distributed (Figure 6D), newly synthesized Dscam[TM1]::GFP was consistently located to dendrites (Figure 6B). These observations suggest involvement of selective transport in targeting Dscam[TM1] specifically to the dendrites.

We next co-expressed dominant-negative Glued to determine how compromised dynein/dynactin function might affect the sorting of newly synthesized Dscam[TM1]::GFP. Analogous transient co-induction did not alter the dendritic distribution of Dscam[TM1]::GFP (data not shown, similar to Figure 6E). However, an acute prolonged induction revealed a requirement for dynein/dynactin in the continuous restriction of Dscam[TM1] to the dendrites (Figures 6F–6H). Organisms carrying both *UAS-Dscam[TM1]::GFP* and *UAS- Gl^Δ^* were reared at 18°C until the wandering larval stage when they were subjected to a half-hour heat shock at 38°C followed by continuous incubation at 30°C. Interestingly, starting around six hours after heat shock, Dscam[TM1]::GFP gradually misdistributed into the axons (Figure 6M). Dscam[TM1]::GFP was first detected in the proximal region of peduncles (Figure 6F), then present in the beginning of axonal lobes (Figure 6G), and, by 12 hours after heat shock, located throughout the entire axon lobes (Figure 6H). By contrast, in the absence of dominant-negative Glued, Dscam[TM1]::GFP remained restricted to the MB calyces even after 28 hours of continuous induction (Figures 6I–6L). These results indicate that the misdistribution was not due to excessive expression of Dscam[TM1]::GFP, but rather owing to disruption of dynein/dynactin function by dominant-negative Glued.

Two possible scenarios may underlie the time course of mislocalization. First, several hours of continuous induction might be needed to express enough truncated Glued for blocking dynein/dynactin function. Second, dynein/dynactin could be dispensable to the selective transport of Dscam[TM1] from cell bodies to dendrites, and specifically involved in removing any mistargeted Dscam[TM1] out of the axons. In this case, blocking dynein/dynactin function should not affect the initial dendritic targeting of Dscam[TM1]::GFP, but would compromise the ability of neurons to promptly move Dscam[TM1]::GFP from the axon ‘hillock’ back to the somatodendritic region.

To determine if such a protracted process of misdistribution occurred as a consequence of slow accumulation of dominant-negative Glued, we further examined how increasing the dosage of dominant-negative Glued affects the misdistribution process [52]. We could drastically shorten the time to detect mCD8::GFP in TARGET by doubling the copy number of UAS transgene (data not shown). If induction of dominant-negative Glued was the rate-limiting factor, increasing the dosage of truncated Glued should accelerate the onset of mistargeting. As the copy number of *UAS- Gl^Δ^* transgene was increased to two and even three, we did not detect any change in the profile of the slow-onset, gradual accumulation of Dscam[TM1]::GFP in the MB axons (Figure 6M). We did not see any reduction in the level of induction of *UAS-Dscam[TM1]::GFP* either, reassuring an ample supply of GAL4 even in the presence of four UAS transgenes. These results indicate that the induction of dominant-negative Glued was not limiting the misdistribution process.

In summary, these pulse-induction experiments ascribe a primary role to the mechanism of selective transport in the dendritic targeting of Dscam[TM1]. This explains why newly synthesized Dscam[TM1] can be promptly located to dendrites, showing no evidence for incidental mistargeting. However, trace amounts of Dscam[TM1] may distribute to axons. It never accumulates to a detectable level in axons with intact dynein/dynactin function. Dynein/dynactin mediates retrograde axonal transport which apparently plays a scavenger role in the restriction of Dscam[TM1] to dendrites.

## Discussion

Multiple lines of evidence indicate that the dynein/dynactin complex has an important function in maintaining proper distribution of dendritic Dscam in MB neurons. First, mutations in three components (*Lis1*, *Dmn* and *p24*) of the dynein/dynactin complex were recovered based on mislocalization of dendritic Dscam through a MARCM-based genetic mosaic screen (Figures 2A–2C). Second, silencing other components of the complex with RNAi also resulted in mistargeting of dendritic Dscam to axons (Figures 3B–3G). Third, disrupting dynein/dynactin function with dominant-negative Glued reproduced the mislocalization phenotype (Figure 4B). Further, newly synthesized Dscam[TM1] was preferentially targeted to dendrites (Figure 6B). Interestingly, compromising dynein/dynactin function did not affect the targeting from cell bodies to dendrites but disrupted the continuous exclusion of dendritic Dscam from axons (Figures 6E–6H, and 6M). Altogether, our findings show that dynein/dynactin normally acts to prevent Dscam[TM1] from entering axons by retrograde axonal transport.

Acute induction by TARGET revealed two mechanisms underlying the dendritic distribution of Dscam[TM1]. Newly synthesized Dscam[TM1] was largely excluded from axons, suggesting directed dendritic targeting and the involvement of selective transport in the dendritic distribution of Dscam[TM1]. Though dynein/dynactin is essential for restricting Dscam[TM1] to dendrites, knocking down dynein/dynactin function did not disrupt the directed dendritic targeting. This leads us to believe that dynein/dynactin is required for preventing dendritic Dscam from misdistributing into axons. When dynein/dynaction function was compromised, newly synthesized Dscam[TM1] remained consistently targeted to dendrites but later leaked into axons. Dendritic Dscam gradually filled the axons; and it took about six hours for Dscam[TM1] to reach the axon termini. This protracted process of mislocalization suggests that dendritic Dscam passively leaks into the axons, and that dynein/dynactin-mediated retrograde axonal transport normally acts to rapidly move leaked Dscam[TM1]-containing vesicles out of the axons. In summary, these phenomena not only demonstrate a dynein-dynactin-independent mechanism of selective transport that preferentially targets Dscam[TM1]-containing vesicles to dendrites, but also implicate the involvement of retrograde axonal transport in preventing accumulation of Dscam[TM1] in axons. These two independent mechanisms act together to ensure restriction of dendritic Dscam to the dendrites.

**Figure 7:**
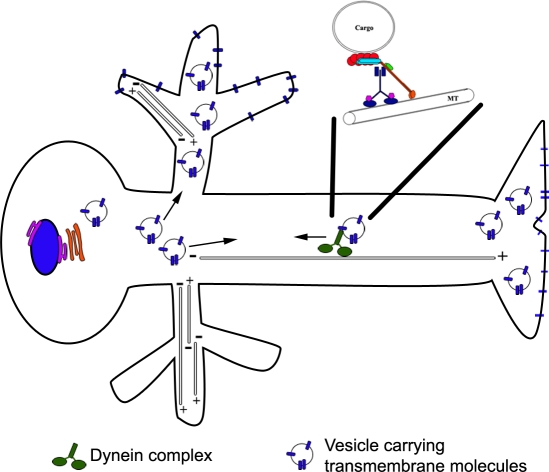
Multiple mechanisms govern the dendritic distribution of Dscam[TM1]. Dscam[TM1]-containing cargos are primarily targeted to dendrites via a dynein/dynactin-independent process. In addition, they are effectively excluded from the axons by dynein/dynactin-mediated retrograde axonal transport.

Although the dynein/dynactin complex is essential for maintaining dendritic distribution of Dscam[TM1], our results do not reveal whether mislocalized Dscam[TM1] is on the plasma membrane or in vesicles inside the cytoplasm. It is possible that dendritic Dscam passively leaks into axons either through membrane diffusion or mistargeting of vesicles. Since blocking endocytosis with temperature-sensitive *shibire* mutant showed no obvious effect on Dscam dendritic distribution (data not shown; [53]), we favor the model that dynein/dynactin acts to prevent axonal accumulation of Dscam[TM1] by actively moving mistargeted Dscam[TM1]-containing vesicles out of axons by retrograde axonal transport (Figure 7).

However, dynein/dynactin is not routinely needed for excluding dendritic proteins from the axons. Since no biological process can be carried out with absolute fidelity, it is conceivable that dendritic molecules of most kinds may accidentally leak into the axons. Some salvage mechanism(s) should exist for actively clearing mislocalized molecules to prevent any significant accumulation in the wrong places. One of the possibilities is that dynein/dynactin mediates retrograde axonal transport and can serve as a general mechanism for removing dendritic molecules out of axons. This hypothesis remains to be tested thoroughly. Nonetheless, blocking dynein/dynactin function did not affect the distribution of two other dendritic markers we checked. Nod-β-gal is a reliable minus-end reporter of microtubules, and misdistribution of Nod-β-gal in MB axons has been shown in *short stop* mutant clones, in which microtubule polarity is perturbed [47]. Absence of Nod-β-gal from the axons of dynein/dynactin mutant neurons demonstrates that the microtubules in axons remained uniformly polarized with minus ends pointing toward cell bodies, and rules out the possibility that dendritic Dscam became mislocalized due to abnormal microtubule organization. As to Rdl-HA, which, like Dscam[TM1], is a membrane protein, a lack of effect on its somatodendritic distribution indicates that dynein/dynactin is selectively involved in preventing dendritic Dscam from leaking into the axons. Diverse mechanisms may be utilized to efficiently clear different dendritic proteins in axons.

Regarding the mechanism(s) of selective transport, directed dendritic targeting apparently requires motor proteins that selectively move cargos toward the dendrites. Since dendrites, but not axons, carry microtubules with minus ends pointing away from cell bodies, potential candidates that underlie directed dendritic targeting include all minus-end-directed microtubule motors. Notably, dynein/dynactin is dispensable to the initial dendritic targeting of Dscam[TM1] or the continuous dendritic restriction of Rdl, arguing against any critical role for minus-end-directed dynein/dynactin in transporting cargos into the dendrites. Other microtubule motors that might support such directional movement include dendrite-specific plus-end-directed motors (e.g. KIF17 and KIF21B), though it remains mysterious how a plus-end-directed motor can be well restricted to dendrites [54], [55]. In theory, forward genetic mosaic screens will ultimately allow us to uncover the diverse mechanisms of dendritic protein targeting. Encouragingly, we have obtained mutants that exhibit different mislocalization phenotypes, further characterization of which should shed additional light on neuron polarity and its underlying cellular/molecular mechanisms. Notably, in DC-B9 mutant clones, mistargeted Dscam[TM1]::GFP existed abundantly in the MB peduncle, preferentially accumulated at the end of the peduncle, but never extended into the axon lobes. This intriguing phenotype suggests presence of distribution barriers not only in the beginning of axons but also at the junction between the proximal axon domain (peduncle) and the distal axon segment (lobe), and implies another possible mechanism for restricting Dscam[TM1] to the dendritic membrane.

Furthermore, the functional roles of each subunit of the dynein/dynactin complex have not been fully determined [13]. Although several studies of the dynein light chains in mammalian cells indicate that dynein subunits can be functionally specialized [56], studies in *Drosophila* show that strong loss-of-function mutations in different dynein/dynactin subunits show extensive overlap in the resulting mutant phenotypes [47], [57]. Our data indicate that *Lis1*, *Dmn*, *Glued*, *p24*, *p25*, *Dhc64C*, *Dhc62B*, and *Dlc90F* all participate in the complete function of dynein/dynactin complex in maintaining dendritic distribution of Dscam. This result supports the idea that all the dynein/dynactin subunits work together to fulfill its diverse functions, and loss of any subunits may result in different degrees of similar dynein/dynactin-dysfunctional phenotypes.

With respect to Dscam targeting motifs, we have reported that the cytoplasmic juxtamembrane domain of Dscam may dictate its TM-dependent subcellular localization [27]. However, further structure-distribution analysis only allowed us to locate an axonal targeting motif to the cytoplasmic juxtamembrane region of TM2, leaving its dendritic targeting motif(s) still undetermined (unpublished results). In addition, we could not determine using the same system whether any of the mutants recovered here also affects the axonal targeting of Dscam[TM2], since transgenic Dscam[TM2] becomes uniformly distributed upon overexpression following an analogous induction. The involvement of multiple mechanisms in targeting specific Dscams to specific neuronal domains further supports the notion that Dscam isoform compositions in the dendrites versus axons of the same neurons need to be independently regulated, elucidation of the physiological significance of which promises to shed new light on how the brain develops and operates.

In summary, we have uncovered a scavenger mechanism for maintaining dendritic distribution of Dscam[TM1] and provide an in vivo model to study neuron polarity and differential protein targeting. On top of the many known functions of dynein/dynactin (including mitosis, vesicular transport, retrograde signaling, neuronal migration), dynein/dynactin helps restrict certain dendritic proteins to the somatodendritic domain of neurons by preventing them from spreading into the axons. Notably, multiple independent mechanisms act together to locate Dscam[TM1] to dendrites; and diverse mechanisms are utilized to target different dendritic proteins to the dendrites.

## Materials and Methods

### Generation of *UAS-mCD8::RFP*

The monomeric red fluorescence protein (*mRFP*) open reading frame [58] was amplified by PCR and was cloned into the mCD8-comtaining pBS [29] with BamHI and XbaI as the cloning sites, generating a new ORF with *mRFP* fused in frame to the 3′ of mCD8. Then, *mCD8::RFP* was subcloned into *pUAST* [59] with XhoI and XbaI as the cloning sites. *pUAST-mCD8::RFP* transgene was introduced into the fly genome via P element-mediated germline transformation by Genetic Services Inc., MA.

### Fly Stocks and Crosses

For creation of MARCM clones, we crossed *UAS-mCD8::RFP*; *hs-FLP*, *FRT^G13^*, *tubP-GAL80/CyO,Y* to either wildtype or mutagenized *UAS-Dscam[TM1]::GFP*, *FRT^G13^*, *GAL4-201Y/CyO,Y*. *UAS-Nod-β-gal* [46] or *UAS-Rdl-HA* [44] was incorporated on third or X chromosomes, respectively, for examining their distribution in MARCM clones.

For acute induction by TARGET system, we crossed *UAS-Dscam[TM1]::GFP* or *UAS-mCD8::GFP* to *tubP-GAL80^ts^*; *tubP-GAL80^ts^*; *GAL4-OK107*. *UAS-dominant-negative Glued*, *P[UAS-Gl^Δ84^]*, was used to block dynein/dynactin function [42]. To increase copy numbers of *P[UAS- Gl^Δ^]*, we generated another two insertion lines on third chromosome (*UAS-Gl^Δ008m^* and *UAS-Gl^Δ020m^*) by hopping out *P[UAS-Gl^Δ84^]* from second chromosome.

Other flies stocks collected for this study include *Dmn^k16109^/CyO* (BL-11159), *l(2)06496/CyO* (BL-12316), *Lis-1^k13209^/CyO* (BL-11072), *tubP-GAL80^ts^;Tm2/Tm6B* (BL-7019), *noc/CyO; tubP-GAL80^ts^* (BL-7018), and *RNAi* lines from VDRC stock center (Dietzl et al., 2007), including *CG8446RNAi* (23139), *Lis1RNAi* (6216), *DmnRNAi* (23728), *p25RNAi* (8058), *Dhc64CRNAi* (28054), *Dhc62BRNAi* (48153) and *Dlc90FRNAi* (31750).

### MARCM-based Genetic Screens and Analysis of MARCM Clones

Chemical mutagenesis was conducted in the UAS-Dscam[TM1]::GFP, FRT^G13^, GAL4-201Y male flies using standard procedure with an EMS concentration of 40 mM [60]. Individual male progeny derived from the mutagenized flies were then crossed with mCD8::RFP; hs-FLP, FRT^G13^, tubP-GAL80 for MARCM analysis of MB clones. To induce mitotic recombination, newly hatched larvae were heat shocked in a 38°C water bath for one hour and then returned to 25°C. The central nervous systems from wandering third instar larvae were dissected out, fixed and immunostained as previously described [29]. Protein expression was detected by the rabbit anti-GFP Ab (1∶300, Molecular Probes) and MB lobes were labeled by the 1D4 mAb (1∶80). Immunofluorescent signals were collected by confocal microscopy and then processed using Adobe Photoshop to normalize and exclude the background neurons.

### Deficiency Mapping and Complementation Testing

Following screening, the homozygous lethal mutants were mapped initially by crossing to the second chromosome deficiency kit, provided by the Bloomington Drosophila Stock Center. We performed further fine scale mapping with smaller deficiencies to define the minimal regions containing the lethal mutations. Lines mapped to the similar regions were placed in complementation groups by the complementation testing. Eventually, we tested candidate genes in these regions with available lethal mutant lines from Bloomington.

### Acute Induction of UAS-transgenes by TARGET system

Larvae carrying two copies of *tubP-GAL80^ts^* were cultured at the permissive temperature of 18°C since embryogenesis in order to repress GAL4-mediated transcription [50]. Wandering larvae were shifted to 38°C for 30 min, followed by incubation at the non-permissive temperature of 29°C for various periods.
